# Analysis of the Broiler Chicken Dead-on-Arrival (DOA) Rate in Relation to Normal Transport Conditions in Practice in Germany

**DOI:** 10.3390/ani14131947

**Published:** 2024-06-30

**Authors:** Julia Gickel, Christian Visscher, Nicole Kemper, Birgit Spindler

**Affiliations:** 1 WING (Science and Innovation for Sustainable Poultry Production), University of Veterinary Medicine Hannover, Foundation, 49456 Bakum, Germany; christian.visscher@tiho-hannover.de (C.V.); nicole.kemper@tiho-hannover.de (N.K.); 2 Institute for Animal Nutrition, University of Veterinary Medicine, Foundation, 30173 Hannover, Germany; 3 Institute for Animal Hygiene, Animal Welfare and Farm Animal Behavior, University of Veterinary Medicine Hannover, Foundation, 30173 Hannover, Germany; birgit.spindler@tiho-hannover.de

**Keywords:** slaughterhouse, broiler, transport, DOA, influencing factors, duration, distance, temperature

## Abstract

**Simple Summary:**

The dead-on-arrival (DOA) rate is a good indicator of the quality of broiler chicken transport. This evaluation investigated factors that might have an influence on the DOA rate. Therefore, data from the transport of broilers in Germany between January 2022 and May 2023 were analyzed. The results showed no influence on the DOA rate of the amount of animals per transport, the duration and the distance of the transport, the stocking density, and the average daily temperature. Transport between 11:00 and 17:00 and in fall tended to have a higher DOA rate.

**Abstract:**

In total, around 631 million broilers were slaughtered in Germany in 2022. This evaluation included data of approx. 198 million broilers of different ages and breeds that were transported in Germany in 2022 (31% of all cases of broiler chicken transport in 2022). The aim of this study was to analyze German broiler chicken transport (*n* = 14,054) to the slaughterhouse between January 2022 and May 2023 with regard to the dead-on-arrival (DOA) rate and the possible influencing factors. Therefore, the relation between the total amount of animals per transport, the duration and distance of the transport, the planned stocking density in the transport cages, the average daily temperature and time of day and season of the transport as well as the DOA rate were statistically evaluated. The results showed a mean DOA rate of 0.09% (SD 0.09). Transport conducted at midday (11:00 to 17:00) showed higher DOA rates (*p* < 0.05) than transport at other times (day split into 6 h intervals). The highest mean DOA rate (0.10%) was found in the fall, followed by the winter, while transport in the spring and summer resulted in the lowest DOA rate (*p* < 0.05). All in all, the relatively low DOA rate (%) in Germany indicates the good standard of their broiler transport compared to available data from research in other European countries.

## 1. Introduction

In 2022, about 631 million broilers were slaughtered in Germany, and the preliminary data for 2023 indicate a similar level [1]. It can be assumed that approximately the same number of animals were transported to the slaughterhouse in both years. The transport of animals is regulated in accordance with European Union (Council Directive (EC) 1/2005) and national law (Animal Welfare Transport Ordinance—TierSchTrV, 2009) [2,3]. These regulations deal with requirements concerning the stocking density and duration of transport and the conditions of transportation cages, for instance. It is known that the catching and loading of the animals for transport, and also the transport itself, may cause stress for the animals [4,5]. The amount of dead animals (dead on arrival, DOA) is used as a first indication to identify pre-slaughter welfare issues [6].

The European Food Safety Authority (EFSA) stated in its scientific opinion on ‘Welfare of domestic birds […] transported in containers’ that DOA rates above 0.1% should be investigated [7]. In Germany, the Board of Trustees for Technology and Construction in Agriculture e.V. (KTBL) published a target range of 0.05% and an alarm range of 0.3% [8]. Both should be used as indicators for farmers to evaluate the actual status of animal welfare on their farms [8]. The implementing provision of the national law for Lower Saxony (2014) published a target DOA rate of 0.5%. If a specific transport exceeds this threshold, the slaughterhouse is obliged to report it to the veterinary authorities, which are allowed to initiate follow-up controls of this transport [9].

Comprehensive and recent data about DOA rates are partly available from different European countries, and some of them describe factors influencing the DOA rate. In general, the published mean DOA rates range from 0.126% (in the United Kingdom, 2005 [10]) to 0.46% (data from 2000 and 2001, focusing on Dutch and German flocks slaughtered in the Netherlands [11]).

In the United Kingdom, higher DOA rates were detected between June and August [10]. In the Netherlands, a longer transport time and a higher stocking density led to increased DOA values [11]. A study with data from 1997 to 2004 from the Czech Republic noticed that a shorter distance of transport resulted in fewer dead animals [12]. In the latter data, the highest DOA values were found between June and August and between December and February. A large Italian study with 1226 million broiler chickens (data from August 2001 to July 2005, mean DOA rate: 0.35%) confirmed higher DOA rates during summer [13]. A study from France [14] published a DOA rate of 0.18%, showing the influence of the density in crates (more space allowance being associated with less mortality) and climatic conditions (rain and wind being associated with more DOA). More recent European studies were published with data from Belgium from 2013/2014 (DOA: 0.3% [6]) and with data from Spain from 2015/2016 (DOA: 0.187% [15]). In the Spanish study, a maximum temperature above 21.5 °C, a high distance of transport, a long time of transport, and a high average weight were rather related to a high level of DOA [15].

In 2006, a Belgian publication investigated possible reasons for DOA, finding that caution in loading and the general good condition of the animals is the best way to prevent dead animals during transport [16]. A more recent study also stated that lung congestion is the most frequent reason for dead animals during transit [17].

In Germany, comprehensive data on the transport of broiler chickens to the slaughterhouse are missing, as these were previously only used for internal control purposes and have not been collected from the companies for research purposes. This evaluation has succeeded in collecting and harmonizing these data in order to gain a meaningful insight into the German transportation of broilers. The aim of this study was to analyze data on the transport of broiler chickens under usual transport conditions in practice in Germany conducted between January 2022 and May 2023 with regard to the DOA rate and to identify possible influencing factors. For this purpose, the relationship between the number of transported animals, the distance and duration of the transport, the stocking density in the truck, the average daily temperature and the season of the transport, the time of transport and loading as well as the origin of the transport and the DOA rate was investigated. In addition, it was investigated whether transports with a short/long duration, a specific season or a specific average daily temperature were postponed to a specific time in order to avoid high DOA rates, as indicated by the results of previous smaller studies.

## 2. Materials and Methods

### 2.1. Description of the Data Set

**Figure 1:**
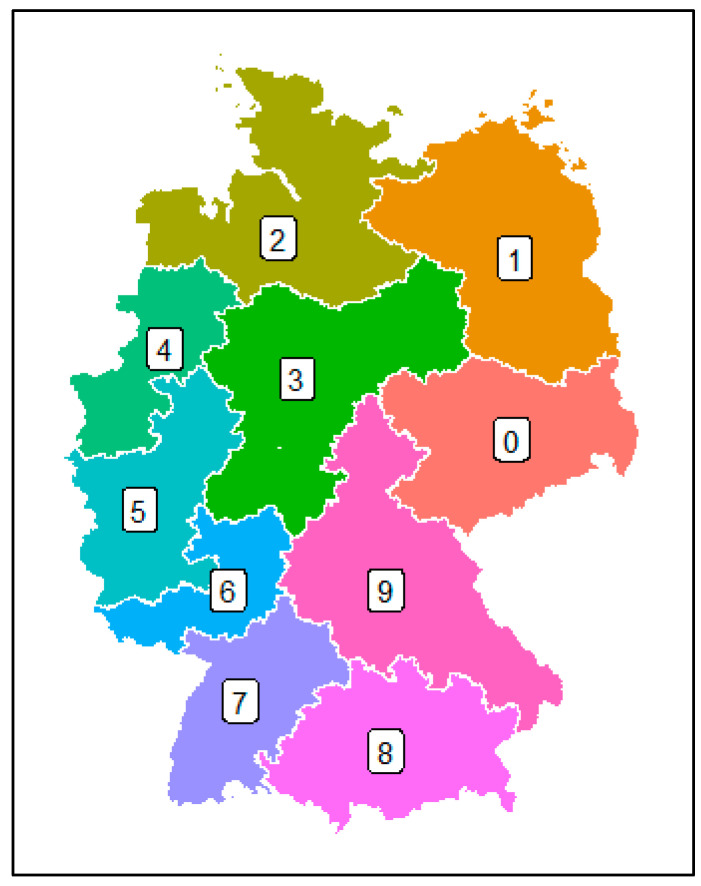
Overview of Germany, with areas classified based on the first digit of the zip code.

**Figure 2:**
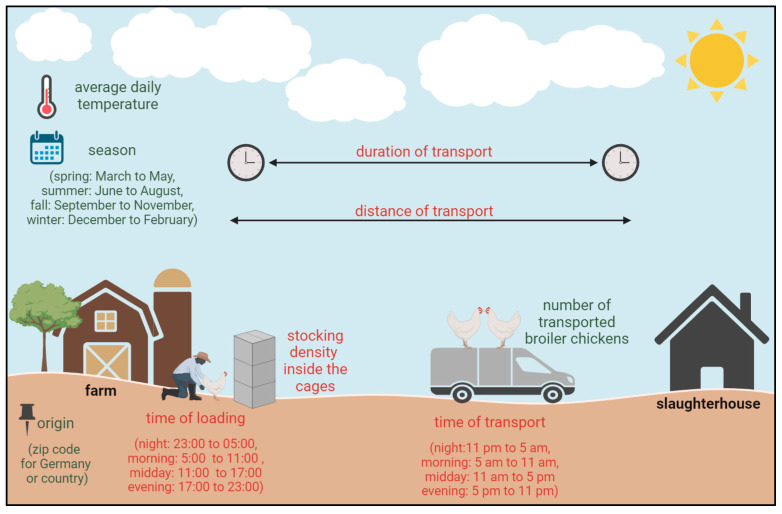
Overview of the evaluated factors influencing the dead-on-arrival (DOA) rate; planned data displayed in red, real data displayed in green (created with BioRender.com).

In total, 14,054 broiler chicken transports between January 2022 and May 2023 (3 January 2022 to 9 May 2023), with about 210 million broilers, were included. These data were provided by several companies with access to databases containing data from already conducted transports. For each transport, the data set included the DOA rate, defined as the percentage of animals per transport unit that were dead on arrival at the slaughterhouse. In this case, the transport unit was not the single truck but all the animals loaded at this destination. The other included parameters were information about the number of transported broiler chickens per transport unit, the actual date of the transport, planned start and end time of the loading and transport, intended duration (min) and distance (km) of the transport, and the zip code (for transports with a German origin only) or the country of origin (Figure 1). About 85% of the data on conducted transports also included the planned stocking density inside the transport cages in the truck (kg per cm^2^, calculated with the average live weight at loading and the size of the cages). The loading times and the durations and distances of the transports were provided as planned data for the respective transport, while in contrast, the arrival at the slaughterhouse, date of transport, number of broilers, and origin of transport were real data (Figure 2). Possible changes, for example, because of a lot of traffic, delays during the loading process, or general rearrangements were not considered. Transports with known unplanned incidents, such as failure of technology on the truck, were excluded from this data set. If either the distance or the duration of a specific transport was missing, a mean speed of 60 km per hour was assumed to calculate the missing value.

The date of each individual transport was used to find out the average daily temperature recorded in Northern Germany (historic data from Bremen, Germany) by the German Meteorological Service (DWD).

The date was also used to categorize each transport into a certain season. Therefore, all the transports in March to May were considered as “spring”, all the transports from June to August as “summer”, all the transports from September to November as “fall”, and all the transports between December and February were classified as “winter”. To distinguish the times of loading and transport, for each of them, the end time was used to classify them into “night” (23:00 to 05:00), “morning” (05:00 to 11:00), “midday” (11:00 to 17:00), or “evening” (17:00 to 23:00).

All the parameters were classified as planned or real data (Figure 2).

### 2.2. Statistics

The influence of the number of animals per transport unit, the duration and distance of the transport, the mean daily temperature, and the stocking density on the DOA rate was evaluated using a correlation analysis (according to Pearson). Cohen’s guideline (1988) was used to assess the correlation as follows: <0.1 = no relation, ≥0.1 and <0.3 = small relation, ≥0.3 and <0.5 = medium relation, and ≥0.5 = high relation [18]. Differences in the season, loading time, time of transport, first digit of the zip code or country on the level of the DOA rate were evaluated using an ANOVA with a post hoc Tukey’s test with a significance level of 0.05. If boxplots are shown, they include the first and third quartile as the frame of the box, the mean marked with a line in between the box, and values below the first quartile and above the third quartile marked with dots. To describe the distribution of the data, the 95% confidence interval and/or the standard deviation (SD) were calculated. A possible influence of different factors on the loading time and the time of transports was furthermore studied by focusing on the percentage distribution.

All the calculations were performed using R Statistical Software (v4.3.0 [19]).

## 3. Results

### 3.1. Distribution of the Level of the DOA Rate (%)

**Figure 3:**
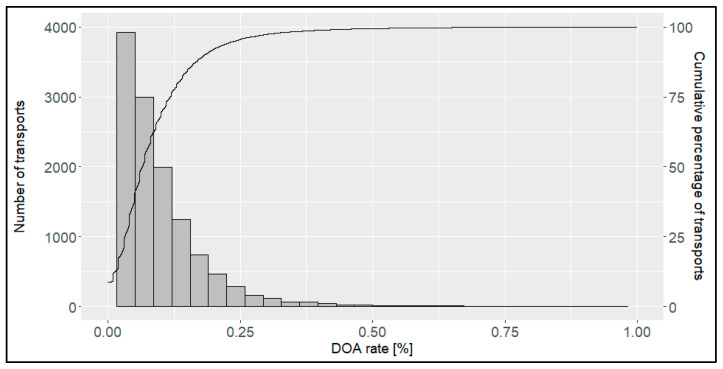
Number of transports (bars) and cumulative percentage (line) of transports per dead-on-arrival (DOA) rate [%]) (*n* = 14,054 broiler transports).

Most transports showed a low DOA rate (Figure 3). The lowest DOA rate (%) was 0.00 and the highest 3.36, with a mean DOA rate (%) of 0.09. In total, 0.09% of all the transports reached the slaughterhouse without any dead broilers. In 50% of all the transports, the DOA rate (%) was below 0.07; in 75% of all the transports, it was below 0.12; and in 90% of all the transports, it was below 0.19. Only 0.06% of all the transports were above the rate of 1%. A DOA rate of below 0.5% was reached by 99.4% of all the transports (Table 1).

### 3.2. Factors Influencing the DOA Rate (%)

The following sections describe the results for each influencing factor. In each figure, the y-axis is set to the boundaries of 0% at the lower and 1.5% at the upper end. Outliers above this level (very few; see Section 3.1) are therefore not visible in the figures.

#### 3.2.1. Relation between the Number of Transported Animals and the DOA Rate (%)

**Figure 4:**
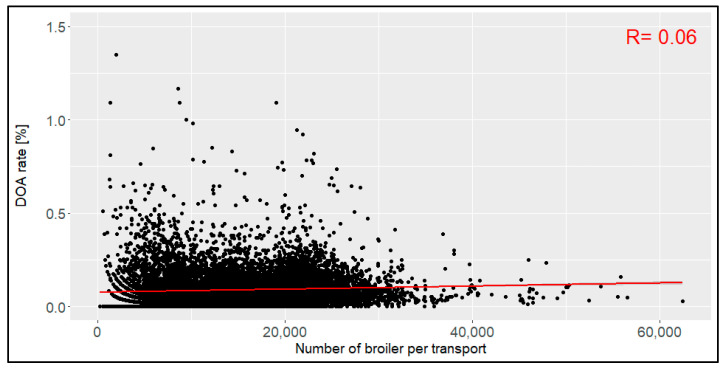
Relation between the number of broilers per transport unit and the dead-on-arrival (DOA) rate [%], including the correlation coefficient (R) (*n* = 14,054 broiler transports).

The number of transported broilers per transport unit ranged between 242 and 62,377 animals (Figure 4), with a mean of 14,936 broilers (SD: 7260). The confidence interval was 706 to 29,165. With a correlation coefficient of 0.06, no relation was found between the number of broilers per transport unit and the DOA rate.

#### 3.2.2. Relation between the Distance or Duration of Transport and the DOA Rate (%)

**Figure 5:**
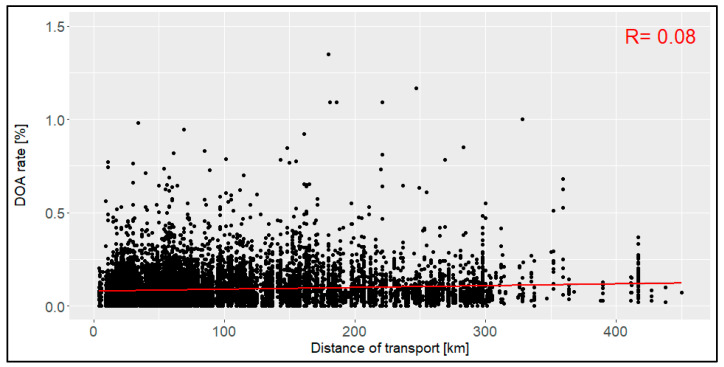
Relation between the distance of the transport [km] and the dead-on-arrival (DOA) rate [%], including the correlation coefficient (R) (*n* = 14,054 broiler transports).

**Figure 6:**
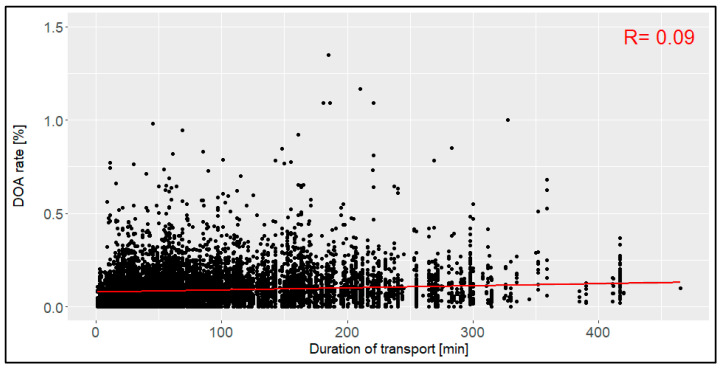
Relation between the duration of the transport [min] and the dead-on-arrival (DOA) rate [%], including the correlation coefficient (R) (*n* = 14,054 broiler transports).

The transport distance between the loading location and the slaughterhouse ranged between 4.0 km and 450.0 km (Figure 5), with a mean of 95.3 km (SD: 81.0 km). This led to a duration of between 1 min and 465 min and a mean of 92 min (SD: 78 min), respectively (Figure 6). With correlation coefficients of 0.08 and 0.09, no relation was found between the distance or the duration and the DOA rate. The confidence intervals included all the data below 254 km and below 245 min.

#### 3.2.3. Relation between the Stocking Density inside the Truck and the DOA Rate (%)

**Figure 7:**
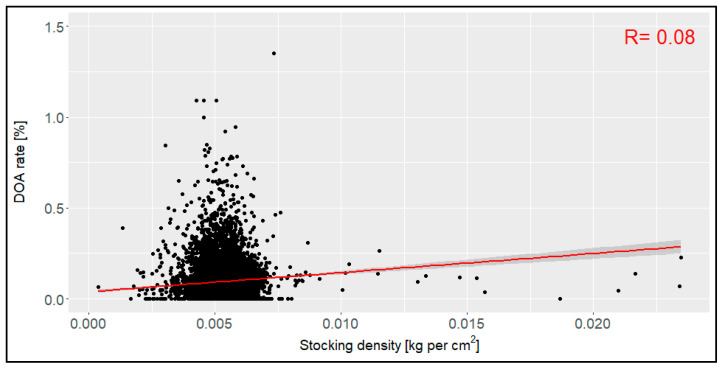
Relation between the stocking density [kg per cm^2^] and the dead-on-arrival (DOA) rate [%], including the correlation coefficient (R) (*n* = 11,966 broiler transports).

The stocking density in all 11,966 transports with data available concerning this factor differed between 0.0004 kg per cm^2^ and 0.023 kg per cm^2^, with a mean value of 0.005 kg per cm^2^ (Figure 7). The correlation coefficient was 0.08, indicating that there was no relation between the stocking density and the DOA rate. The confidence interval was 0.0036 to 0.0068 kg per cm^2^.

#### 3.2.4. Relation between the Average Daily Temperature in Northern Germany and the DOA Rate (%)

**Figure 8:**
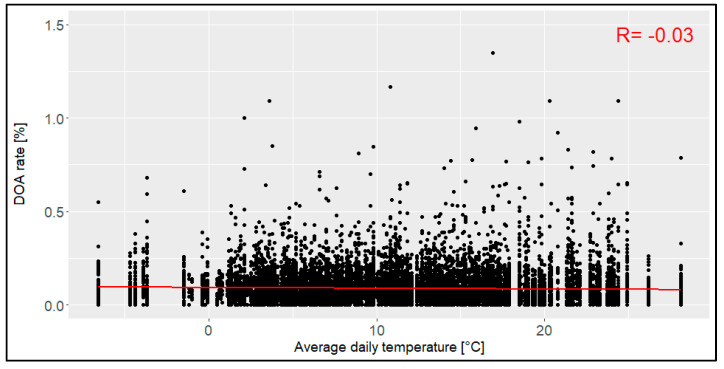
Relation between the average daily temperature [°C] and the dead-on-arrival (DOA) rate [%], including the correlation coefficient (R) (*n* = 14,054 broiler transports).

The average daily temperature in Northern Germany for all the transports differed from -6.6 °C to 28.1 °C, showing a mean value of 11.0 °C (Figure 8). The confidence interval ranged from -1.8 °C to 24.3 °C. The correlation was 0.03, indicating that there was no relation between the average daily temperature in Northern Germany and the DOA rate. Transports below 0 °C (3.4% of all the transports) and above 25 °C (0.8% of all the transports) were rare.

#### 3.2.5. Relation between the Season of the Transport and the DOA Rate (%)

**Figure 9:**
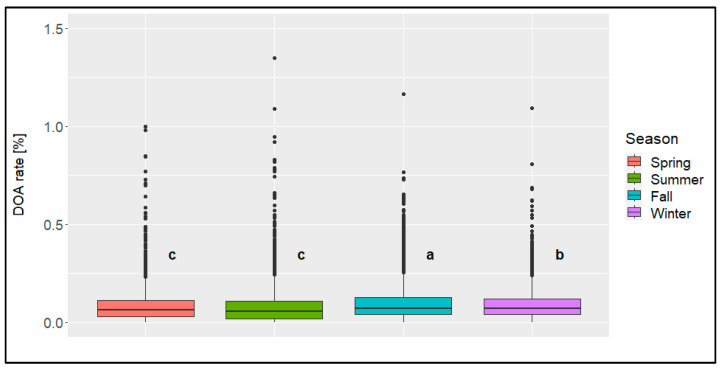
Boxplots of the dead-on-arrival (DOA) rate [%] per season; significant differences (*p* < 0.05) are marked with different letters (*n* = 14,054 broiler transports).

In general, the numbers of transports per season were similar (Table 2). In all the seasons, the minimum DOA rate was 0.00%, while the highest values differed from 1.00% (spring) to 3.36% (summer). The ANOVA results showed that the DOA rate was the highest in fall (significant), followed by the DOA rate in winter (Figure 9). The DOA rates in spring and summer were similar to each other. The boxplots, or rather the location of the first and third quartiles, indicated that most of the transports in all the seasons showed a DOA rate above 0.02% and below 0.13%.

#### 3.2.6. Relation between the Loading Time or the Time of Transport and the DOA Rate (%)

**Figure 10:**
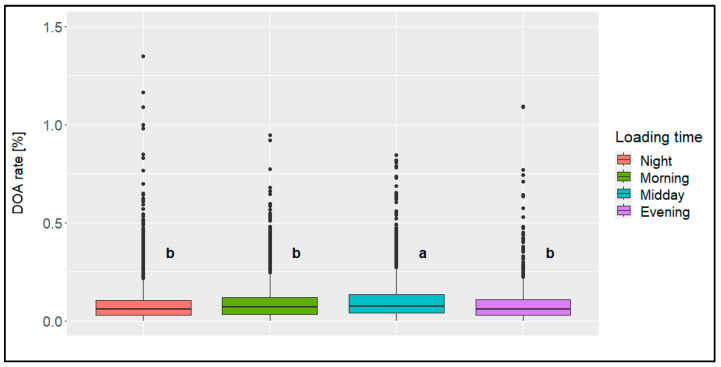
Boxplots of the dead-on-arrival (DOA) rate [%])per loading time (categories: night [23:00 to 05:00], morning [05:00 to 11:00], midday [11:00 to17:00], evening [17:00 to 23:00]); significant differences (*p* < 0.05) are marked with different letters (*n* = 14,054 broiler transports).

**Figure 11:**
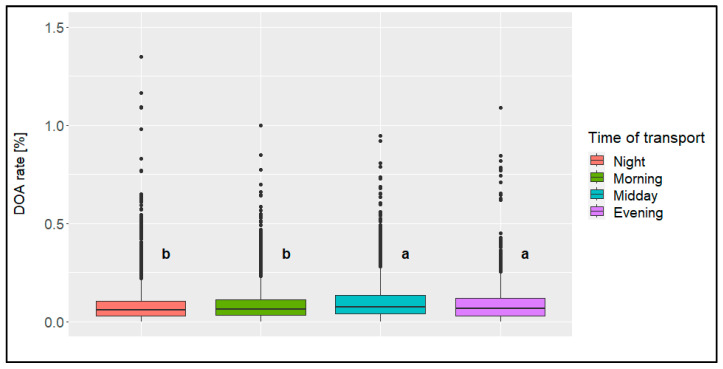
Boxplots of the dead-on-arrival (DOA) rate [%] per time of transport (categories: night [23:00 to 05:00], morning [05:00 to 11:00], midday [11:00 to 17:00], evening [17:00 to 23:00]); significant differences are marked with different letters (*n* = 14,054 broiler transports).

The highest percentage of all the loadings (approx. 37%) and the highest percentage of all the transports (approx. 42%) were conducted in the morning (Table 3 and Table 4), while the lowest percentage was conducted in the evening (7% of all the loadings, 8% of all the transports). The significant (*p* < 0.05) highest mean DOA rate was found concerning the loadings at midday and concerning the transports at midday or in the evening (Figure 10 and Figure 11). For both influencing factors, all the other time categories were similar to each other.

#### 3.2.7. Relation between the Origin of the Transports and the DOA Rate (%)

Nearly all the transports (99.9%) set off from barns in Germany; only 13 transports came from the Netherlands, while 5 transports originated in Belgium. The ANOVA results indicated no influence of the country of origin on the DOA rate. However, these results need to be classified as not expressive due to the distinctly different amount of data per country.

Concerning the transports from Germany only, no data were given for zip codes starting with the digit seven or eight, which mainly include the south of Germany (Table 5, Figure 1). It is also striking that the majority (approx. 50%) came from the region with zip codes starting with the digit four, covering an area in the west of Germany. This region was followed by the region with zip codes starting with the digit two (approx. 30%), covering an area in the north and north-west of Germany. Approx. 14% of all the transports originated from the region with a zip code starting with the digit three (central Germany), while approx. 5% of all the transports could be assigned to the area with zip codes starting with the digit five (west Germany, south of west Germany). The other regions only accounted for approx. 1% of all the transports.

**Figure 12:**
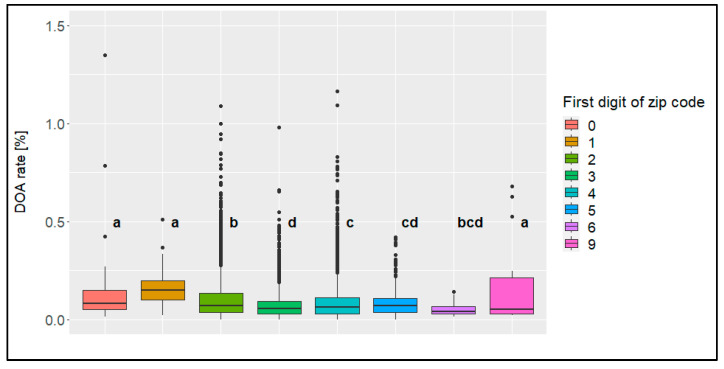
Boxplots of the dead-on-arrival (DOA) rate [%] per the first digits of zip codes, including transports from Germany only; significant differences are marked with different letters (*n* = 14,037 broiler transports).

The regions with zero, one or nine at the beginning of the zip code showed the highest mean values, with significant differences from the other groups (Figure 12). These included the eastern part of Germany. However, since all these regions included low frequencies, the influence of one single transport was higher than in other regions, which can contribute to distorting the results. When focusing on the three regions with larger amounts (zip code starting with the digit two, three, or four), the highest mean value could be found for the region starting with the digit two, followed by the region starting with the digit four, while the region starting with three showed the lowest mean value of these three regions. All of these differences were significant.

### 3.3. Influence of Factors on the Decision Regarding a Loading Time and a Time of Transport

#### 3.3.1. Influence of the Duration of Transport, Season, and Average Daily Temperature on the Loading Time

The loadings for transports of between 100 min and 400 min were mostly conducted during the night, while most loadings for short transports (<100 min) were conducted in the morning (Table 6). For a transport duration of 400 min or more, the highest percentage of loadings was found in the evening. However, concerning transports with less than 200 min of duration, loadings in the evening had the smallest percentage. Above 200 min, loadings at midday had the smallest percentage.

Regardless of the season, loadings in the evening had the lowest percentage (Table 7). The highest percentage of loadings could be found during the night or in the morning, irrespective of the season. From all the season loadings, those in the fall seemed to differ the most from the other seasons, showing a slight trend toward more loadings during the night and fewer loadings at midday.

Concerning the average daily temperature, loadings below 0 °C and above 25 °C were rare (Table 8). In all the categories of average daily temperature, the lowest percentage of loadings occurred in the evening and the highest percentage was found in the morning (excluding 10 °C to 15 °C).

#### 3.3.2. Influence of the Duration of Transport, Season and Average Daily Temperature on the Time of Transport

All the transports below a duration of 400 min were mostly conducted in the morning (Table 9). Between 200 min and 400 min, more than half of the transports may be assigned to this period. Transports equal and above 400 min (*n* = 47) were mostly conducted during the night. Excluding this small group of transports, it seems that short transports (<100 min) tended to be conducted at midday, whereas longer transports were conducted in the evening. In general, transports below 100 min were the largest group (approx. 68%).

Regardless of the season, transports in the morning had the highest percentage and transports in the evening had the lowest percentage (Table 10). Both percentages were nearly stable for all the seasons. When considering the proportion of the remaining percentage, it seems that transports at midday were rarer in the fall than in the winter, while those at night were more frequent.

Concerning the average daily temperature, transports below 0 °C and above 25 °C were rare (Table 11). In all the categories of average daily temperature, the lowest percentage of loadings occurred in the evening, whereas the highest percentage was found in the morning.

## 4. Discussion

The available data for the year 2022, with a total of approx. 198 million broilers, amount to around 31% [1] of all the broiler chicken transports to slaughterhouses carried out in Germany in that year and thus constitute a comprehensive sample. However, by excluding some transports with special incidents, the data set does not represent the entire range of all the transports in Germany. In this evaluation, there was a focus on transports representing a typical “normal transport” without issues. It is therefore to be expected that the overall number of outliers is somewhat higher. According to the EFSA, the DOA rate may serve as an “iceberg indicator for transport conditions” [7] for the welfare of animals during these specific transports, although it is known that the DOA might also be influenced by other factors. The mean DOA rate of 0.09% in this evaluation represents a value below previous European studies with a range from 0.13% (United Kingdom, 2005 [10]) to 0.46% (data from 2000 and 2001, focusing on Dutch and German flocks slaughtered in the Netherlands [11]). This leads to the assumption that broiler chicken transports in Germany have a comparable low DOA rate. On the other hand, it may also be assumed that the excluded transports had a higher DOA rate. It could be possible that unplanned incidents such as the failure of technology on the truck (left out in this evaluation) could have resulted in a higher DOA rate, thus raising the overall mean. This leads to the assumption that this overall mean is not representative of broiler chicken transport in Germany in general but of broiler chicken transports usual in practice that experience no major difficulties. However, in view of the large number of transports already included in the evaluation, it can be assumed that these data would not have had a serious impact on the results of this evaluation regarding the influencing factors.

More than 97% of all the observed transports in this evaluation complied with the target values of TierSchTrV (0.5%) [9] and the alarm value of the KTBL (0.3%) [8] and may therefore be classified as having no severe issues. The target value of the EFSA (0.1%) [7] was only complied with by 67.9% of all the transports, leading to the assumption that this is not a generally feasible standard and cannot be accomplished without adjustments. Even more adjustments in the transport of broiler chickens are probably needed to comply with the target range stipulated by the KTBL [7]. This target DOA rate of 0.05% was achieved by only 37.3% of all the transports within this evaluation.

In our findings, the number of animals per transport unit (i.e., all the animals loaded at any one destination) did not affect the DOA rate. It is important to mention that this value did not reflect the number of animals per truck but the number of animals within a transport and can therefore be equated with a slaughter batch. Neither small nor large loads led to a different DOA rate. As large loads mostly entailed longer loading times and a higher number of loaded animals per worker, it may be assumed that these factors did not affect the DOA rate. Since to the best of our knowledge, no other study has examined this factor, a comparison is not possible.

The distance of transports with a mean of 95 km and the duration of transports with a mean of 92 min did not affect the DOA rate in this study. Prior studies, for example, with data from transports in the Czech Republic (categories from <50 km to >300 km) [12] and with Spanish data (8 km to 119 km, 53 min to 1184 min) [15], however, indicated a clear relation, with longer and long-distance transports leading to higher DOA rates. Although the general ranges of the duration and distance in this evaluation were comparable with those in the two studies above, no reason for this distinction may be stated. It may be assumed that either technology has improved in recent years so that longer and long-distance transports are no longer a problem, or that transports in Germany did not have an issue with the duration and distance of the transports at all.

In this evaluation, the stocking density (range between first and third quantile: 0.0046 and 0.0057 kg per cm^2^) did not affect the DOA rate. However, a previous study from 2004 described that higher stocking densities led to higher DOA rates [11]. As the stocking density in this study was described using birds per compartment without defining the size of a compartment, a direct comparison is not possible. A later study from 2011 published a range between the first and third quartile of between 0.0053 kg per cm^2^ and 0.0062 kg per cm^2^ for their data [14], which is higher overall than the stocking density in our evaluation. It may therefore be assumed that the general mean of the stocking density might have been too low to see the effects that occurred in previous evaluations. Within the framework of the national regulations for the stocking density in the cages, no effects on the DOA rate (%) were to be expected for broiler transports in Germany. To evaluate a possible effect on high or low levels, more data and especially data on the actual (not planned) stocking density should be studied in further evaluations. The average daily temperature in northern Germany (Bremen) did not affect the DOA rate in this evaluation. Concerning this, it is important to note that these temperatures represent mean values for the entire day and therefore do not necessarily reflect the transport phase and certainly not the temperature on the truck itself. In addition, the actual region of transport is not represented in these temperature values, as they are all weather data from a station located in Northern Germany (Bremen). However, they proved to be a good compromise when considering the regions of origin of most of the transports throughout the zip codes. In the Spanish evaluation from 2016, the authors postulated that a maximum temperature above 21.5 °C led to a higher DOA rate [15]. As that study used the maximum daily temperature, while our evaluation used the average daily temperature, these factors are not directly comparable. In this evaluation, transports at a high outside temperature were rare, so it might be assumed that transports were postponed to a colder day if possible. It was also found in this evaluation that the most loadings and transports were conducted during the night or in the morning, meaning that they were not affected by the maximum daily temperature. To study the effects of the temperature on the DOA rate, it is important to link the transport date with the actual temperature at the specific time in the specific region and on the truck to assess all the temperature influences on the animals. However, earlier conditions before loading the animals (e.g., mortality on the farm or the duration of feed withdrawal before loading) also showed a relation with the rate DOA in prior studies [20,21], with indications that these periods should also be considered in an evaluation of DOA rates in broiler chickens. Ritz et al. (2005) also found that the transport itself did not result in higher environmental temperatures for the animals but that temperatures may become higher during the loading due to crowding, delays, or technical issues [21].

No conclusion may be drawn regarding the influence of the country of origin on the DOA rate due to the rarity of data from countries besides Germany. Concerning the region of origin in Germany (zip codes), it may be concluded that regions with a lot of broiler production (with four as the first digit of the zip code) did not show the highest but also not the lowest DOA rate when compared to regions with fewer broilers. Comparisons of North and South Germany are impossible because of missing data from South Germany. Similarly, comparisons of West (with two, four, five, six as the first digits of the zip code) and East (with zero, one, and nine as the first digit of the zip code) would not be meaningful due to the widely different number of data (West: 11,919 transports, East: 113 transports). The region of North-West Germany is known to have more farms on the same area than the region of South-East Germany has [22]. Therefore, it may be assumed that transports from areas with more farms per square kilometer do not result in a higher DOA rate than from regions with fewer farms.

With regard to the seasons, it could not be concluded in our study that the loading and transport times are adapted to these conditions. Fewer transports were generally carried out at night in winter, which may be due to the lower temperature or unsafe road conditions. In general, the mean DOA rate was highest in fall (0.13%) and second highest in winter (0.12%). Both seasons differed significantly from each other as well as from the other two seasons (0.11%). One explanation for this may be the increased incidence of wet weather with storms and rain in fall and winter, which makes it necessary to use tarpaulins on the trucks to protect the animals from external influences. By limiting the exchange of air with the outside, this may also have a negative impact on the air circulation inside the trucks, which could lead to problems in the animals’ cardiovascular system. However, as no data are available that show the climate in the animal area of the trucks, no statement can be made in this regard.

The average DOA rate differed by only 0.01 to 0.02% between the individual times of day. However, it can be ascertained from the available data that loadings at midday led to a significantly higher mean DOA rate when compared to the other times of day, while at all the other times of day, the DOA rates were at a similar level. In addition to the possibly higher temperatures at midday (not covered due to the use of average daily temperature in this evaluation), one reason for this could be the presence of daylight during loading, which cannot always be completely avoided despite management-related factors such as darkening the barn. This could possibly lead to more restless or nervous animals during loading [23]. Shorter transports tended to take place in the morning rather than longer transports. This might be a result of management decisions based on experience values. The same reason might apply to transports in fall (showing the highest DOA rate of all the seasons in this evaluation), where fewer transports were conducted at midday (showing the highest DOA rate of all the time categories in this evaluation).

Even though a wide range of influencing factors were considered in this evaluation, there are still many other possible influencing factors that could also have had a greater or lesser impact on the DOA rate. Factors such as the temperature on the truck, the general behavior of the flocks during fattening (more active or calm), the weight of the animals, the loading process (also with regard to the use of different systems and methods), and the waiting time at the slaughterhouse, etc. were, for example, not studied in this evaluation. However, it may be assumed that these factors were considered for the transports in this evaluation due to the generally low DOA rate. As an earlier study published findings on the relation between the weight of broiler chickens and the DOA rate [15], it would also be interesting to study different rearing systems and breeds. To gain a closer understanding of the factors affecting the DOA rate, it is important to generate more detailed and specific data (for example, concerning the real actual temperature) for as many transports as possible.

## 5. Conclusions

The evaluated data showed no influence of the number of animals, the duration and the distance of the transport, the stocking density, and the average daily temperature on the DOA rate (%). Transports conducted at midday (11:00 to 17:00) were associated with higher rates of DOA than transports at other times. The highest DOA rate was found in the fall, followed by the winter, while transports in the spring and summer resulted in the lowest DOA rate. All in all, the relatively low DOA rate (%) in Germany indicates the good standard of the conducted broiler transports. To gather more knowledge on the welfare of the animals, the evaluation of more factors should be considered. Therefore, further research should focus on more detailed data on different transports, including the weights, rearing systems and breeds, as well as on the actual transport conditions inside the truck and the recording of other animal welfare indicators.

## Figures and Tables

**Table 1:** Percentage of transports and number of transported animals within the data set below published target values [3,7,8] (KTBL = Board of Trustees for Technology and Construction in Agriculture e.V., EFSA = European Food Safety Authority, TierSchTrV = Animal Welfare Transport Ordinance—national law).

Origin of Target Value	Value	Proportion of Transports within This Target Area (%)	Number of Animals within These Transports
KTBL—target range	0.05%	37.3	70 million
EFSA	0.1%	67.9	137 million
KTBL—alarm range	0.3%	97.3	205 million
TierSchTrV	0.5%	99.4	209 million

**Table 2:** Measures of the position and dispersion of the dead-on-arrival (DOA) rate [%] per season (*n* = 14,054 broiler transports).

Measures of Location	Spring	Summer	Fall	Winter
Minimum	0.00	0.00	0.00	0.00
First Quartile	0.03	0.02	0.04	0.04
Median	0.06	0.06	0.07	0.07
Mean	0.08	0.08	0.10	0.09
Third Quartile	0.11	0.11	0.13	0.12
Maximum	1.00	3.36	1.17	1.73
*n*	3688	3657	3437	3272

**Table 3:** Measures of the position and dispersion of the dead-on-arrival (DOA) rate [%] per loading time (categories: night [23:00 to 05:00], morning [05:00 to 11:00], midday [11:00 to 17:00], evening [17:00 to 23:00] (*n* = 14,054 broiler transports).

Measures of Location	Night	Morning	Midday	Evening
Minimum	0.00	0.00	0.00	0.00
First Quartile	0.03	0.03	0.04	0.03
Median	0.06	0.07	0.07	0.06
Mean	0.08	0.09	0.10	0.09
Third Quartile	0.10	0.12	0.13	0.11
Maximum	1.73	0.94	3.36	1.09
*n*	5099	5142	2801	1012

**Table 4:** Measures of the position and dispersion of the dead-on-arrival (DOA) rate [%] per time of transport (categories: night [23:00 to 05:00], morning [05:00 to 11:00], midday [11:00 to 17:00], evening [17:00 to 23:00]) (*n* = 14,054 broiler transports).

Measures of Location	Night	Morning	Midday	Evening
Minimum	0.00	0.00	0.00	0.00
First Quartile	0.03	0.03	0.04	0.03
Median	0.06	0.06	0.08	0.07
Mean	0.08	0.08	0.10	0.09
Third Quartile	0.11	0.11	0.14	0.12
Maximum	1.73	1.00	3.36	1.09
*n*	3809	5872	3203	1170

**Table 5:** Measures of the position and dispersion of the dead-on-arrival (DOA) rate [%] per first digits of zip code (including transports from Germany only) (*n* = 14,037 broiler transports).

Measures of Location	0	1	2	3	4	5	6	9
Minimum	0.01	0.02	0.00	0.00	0.00	0.00	0.01	0.02
First Quartile	0.05	0.10	0.04	0.03	0.03	0.04	0.03	0.03
Median	0.08	0.15	0.07	0.06	0.06	0.07	0.04	0.05
Mean	0.15	0.16	0.10	0.08	0.08	0.08	0.05	0.18
Third Quartile	0.15	0.20	0.13	0.09	0.11	0.11	0.07	0.21
Maximum	1.35	0.51	1.09	0.98	3.36	0.42	0.14	0.68
*n*	40	57	4160	2004	7059	668	32	16

**Table 6:** Distribution of the percentage frequency of different loading times depending on the duration of transport, including the total number of transports per category transport (categories: night [23:00 to 05:00], morning [05:00 to 11:00], midday [11:00 to 17:00], evening [17:00 to 23:00]) (*n* = 14,054 broiler transports).

Duration of Transport	Night	Morning	Midday	Evening	Total (Absolute)
<100 min	30%	40%	23%	7%	9497
100–200 min	44%	32%	18%	5%	2672
200–300 min	57%	27%	5%	11%	1719
300–400 min	49%	30%	8%	13%	119
≥400 min	23%	30%	6%	40%	47
Total (absolute)	3809	5872	3203	1170	14,054

**Table 7:** Distribution of the percentage frequency of different loading times depending on the season of transport, including the total number of transports per category transport (categories: night [23:00 to 05:00], morning [05:00 to 11:00], midday [11:00 to 17:00], evening [17:00 to 23:00) (*n* = 14,054 broiler transports).

Season	Night	Morning	Midday	Evening	Total (Absolute)
Spring	35%	37%	20%	8%	3688
Summer	36%	36%	20%	8%	3657
Fall	38%	36%	19%	7%	3437
Winter	36%	37%	21%	7%	3272
Total (absolute)	3809	5872	3203	1170	14,054

**Table 8:** Distribution of the percentage frequency of different loading times depending on the average daily temperature of the transport, including the total number of transport per range transport (categories: night [23:00 to 05:00], morning [05:00 to 11:00], midday [11:00 to 17:00], evening [17:00 to 23:00]) (*n* = 14,054 broiler transports).

Average Daily Temperature	Night	Morning	Midday	Evening	Total (Absolute)
<0 °C	35%	40%	19%	7%	481
0 to <5 °C	37%	38%	19%	7%	2088
5 to <10 °C	36%	36%	21%	7%	3796
10 to <15 °C	37%	36%	19%	7%	3259
15 to <20 °C	36%	36%	20%	8%	2946
20 to <25 °C	36%	36%	20%	8%	1375
≥25 °C	36%	43%	15%	6%	109
Total (absolute)	3809	5872	3203	1170	14,054

**Table 9:** Distribution of the percentage frequency of different times of transport depending on the duration of the transport, including the total number of transport per category (categories: night [23:00 to 05:00], morning [05:00 to 11:00], midday [11:00 to 17:00], evening [17:00 to 23:00]) (*n* = 14,054 broiler transports).

Duration of Transport	Night	Morning	Midday	Evening	Total (Absolute)
<100 min	25%	41%	25%	10%	9497
100–200 min	32%	39%	21%	7%	2672
200–300 min	31%	52%	14%	3%	1719
300–400 min	21%	51%	21%	7%	119
≥400 min	38%	28%	26%	9%	47
Total (absolute)	3809	5872	3203	1170	14,054

**Table 10:** Distribution of the percentage frequency of different times of transport depending on the season of the transport, including the total number of transports per category (categories: night [23:00 to 05:00], morning [05:00 to 11:00], midday [11:00 to 17:00], evening [17:00 to 23:00]) (*n* = 14,054 broiler transports).

Season	Night	Morning	Midday	Evening	Total (Absolute)
Spring	27%	41%	23%	8%	3688
Summer	27%	42%	22%	9%	3657
Fall	28%	42%	22%	8%	3437
Winter	26%	42%	24%	8%	3272
Total (absolute)	3809	5872	3203	1170	14,054

**Table 11:** Distribution of the percentage frequency of different times of transport depending on the average daily temperature of the transport, including the total number of transports category (categories: night [23:00 to 05:00], morning [05:00 to 11:00], midday [11:00 to 17:00], evening [17:00 to 23:00]) (*n* = 14,054 broiler transports).

Average Daily Temperature	Night	Morning	Midday	Evening	Total
<0 °C	27%	44%	22%	7%	481
0 to <5 °C	27%	42%	24%	7%	2088
5 to <10 °C	26%	42%	24%	9%	3796
10 to <15 °C	29%	41%	22%	8%	3259
15 to <20 °C	27%	42%	23%	9%	2946
20 to <25 °C	27%	41%	22%	10%	1375
≥25 °C	24%	48%	22%	6%	109
Total (absolute)	3809	5872	3203	1170	14,054

## Data Availability

Restrictions apply to the availability of these data. Data were obtained from companies with access to data concerning the transport of broilers and are only available from the authors with the permission of these companies.
